# *Streptobacillus moniliformis* bacteremia in a rheumatoid arthritis patient without a rat bite: a case report

**DOI:** 10.1186/s13104-015-1642-6

**Published:** 2015-11-19

**Authors:** Takahito Nei, Akiko Sato, Kazunari Sonobe, Yoshihiko Miura, Kenji Takahashi, Ryoichi Saito

**Affiliations:** Department of Infection Control and Prevention, Nippon Medical School Hospital, Nippon Medical School, 1-1-5 Sendagi, Bunkyo-Ward, Tokyo, 113-8603 Japan; Department of Orthopedic Surgery, Nippon Medical School, 1-1-5 Sendagi, Bunkyo-Ward, Tokyo, 113-8603 Japan; Department of Clinical Laboratory, Nippon Medical School, 1-1-5 Sendagi, Bunkyo-Ward, Tokyo, 113-8603 Japan; Department of Microbiology and Immunology, Tokyo Medical and Dental University, 1-5-45 Yushima, Bunkyo-ku, Tokyo, 113-8510 Japan

**Keywords:** *Streptobacillus moniliformis*, Bacteremia, Rheumatoid arthritis, Rat bite, Zoonosis

## Abstract

**Background:**

Rat bite fever is a relatively rare infectious disease due to infection with *Streptobacillus moniliformis* or *Spirillum minus* mainly via directs bite by rats, mice, or other rodents. If there is no clear bite history, the diagnosis is difficult or may not be made.

**Case presentation:**

A 72-year-old Asian female with rheumatoid arthritis was admitted for high grade fever and walking difficulty with severe lumbago. Initially, we suspected lumber compression fracture with deterioration of rheumatoid arthritis, but Gram-negative bacilli were isolated from blood culture during hospitalization. The isolated organism was identified as *S. moniliformis* by 16S ribosomal ribonucleic acid (rRNA) sequencing. *S. moniliformis* is well known to be a primary causative organism of rat bite fever, but this patient had no history of rat bite. Had *S. moniliformis* bacteremia not been detected, she might have been treated for rheumatic exacerbation.

**Conclusion:**

We emphasize the importance of performing appropriate microbial culture testing for identifying potential infectious diseases. We also conclude that *S. moniliformis* infection can become established with contaminated vehicle contact alone, not only as a direct result of a bite. We must keep mind that those working in places where rodents breed or are at risk of contact with rats or mice might be at risk for contracting this unusual disease.

**Electronic supplementary material:**

The online version of this article (doi:10.1186/s13104-015-1642-6) contains supplementary material, which is available to authorized users.

## Background

Rat bite fever is a relatively rare infectious disease due to either *Streptobacillus moniliformis* or *Spirillum minus* [1–5]. It presents as an acute or chronic illness with polyarthritis in states such as sepsis and bacteremia [4]. Rat bite fever occurs due to a direct bite by an infected and/or colonized rat, mouse, or other rodent or via intake of contaminated food or milk [4]. Most cases have a history of bites or contact with rodents, but the diagnosis in those without such a history must be based on microbiologic testing. We herewith describe an interesting case of *S. moniliformis* bacteremia without rat bite. This patient had rheumatoid arthritis. Inexplicable joint and bone symptoms closely mimicked those of *S. moniliformis* bacteremia. The diagnosis would thus have been very difficult unless microbial culture tests had revealed the isolate to be *S. moniliformis*.

## Case report

A 72-year-old Asian female presented with high grade fever, 38 °C, and chills which had been present for 8 days prior to admission. She had been followed for rheumatoid arthritis (RA) by the orthopedics department of our hospital for 3 years. At the first visit for febrile episodes, upper respiratory tract infection was diagnosed and she was told to rest and stay in bed. She went home without antipyretics, analgesics or antibiotics. However, she reported worsening lower back pain 3 days after the first visit. Subsequently, she was admitted due to difficulty moving, especially walking, because of severe lower back pain. Furthermore, the presence of a severe inflammatory disease, such as an infection, was suspected based on blood examination results on admission (Table 1).

**Table 1:** Laboratory data on admission

Blood cell counts	
White blood cells	13,300/μL
Neutrophils	95.0 %
Lymphocytes	2.3 %
Red blood cells	367 × 10^4^/μL
Hemoglobin	12.2 g/dL
Platelets	31.1 × 10^4^/μL
Biochemical parameters	
Aspartate aminotransaminase	45 IU/L
Alanine aminotransferase	34 IU/L
Lactose dehydrogenase	202 IU/L
Alkaline phosphatase	1035 IU/L
γ-Glutamyl transferase	239 IU/L
Sodium	136 mEq/L
Potassium	4.5 mEq/L
Chloride	100 mEq/L
Blood urea nitrogen	29.9 mg/dL
Creatinine	1.09 mg/dL
Total protein	6.0 g/dL
Albumin	2.4 g/dL
Serology	
C reactive protein	26.92 mg/dL

Her medical history was unremarkable except for hypertension and RA, reasonably well controlled with low-dose oral steroids and weekly methotrexate. Her familial history was also unremarkable. She neither smoked nor drank alcohol regularly and had no history of caring for animals. Moreover, she had no history of allergies to drugs, foods, or inhaled substances. She ran a dining room in downtown Tokyo. She had undergone dental implant insertions, but had been free of dental caries and periodontal disease for several years.

At the beginning of hospitalization, she was unable to walk because of severe sharp pain, affecting the entire body, including lower back pain which been present prior to admission. She was unable to state definitely where in her body the pain was strongest. She was started on antibiotics (cefazolin 2.0 g/every 8 h) and a nonsteroidal antiphlogistic balm for the pain. Her condition subsequently improved with resolution of the high grade fever spikes, but a low grade fever persisted.

**Fig. 1:**
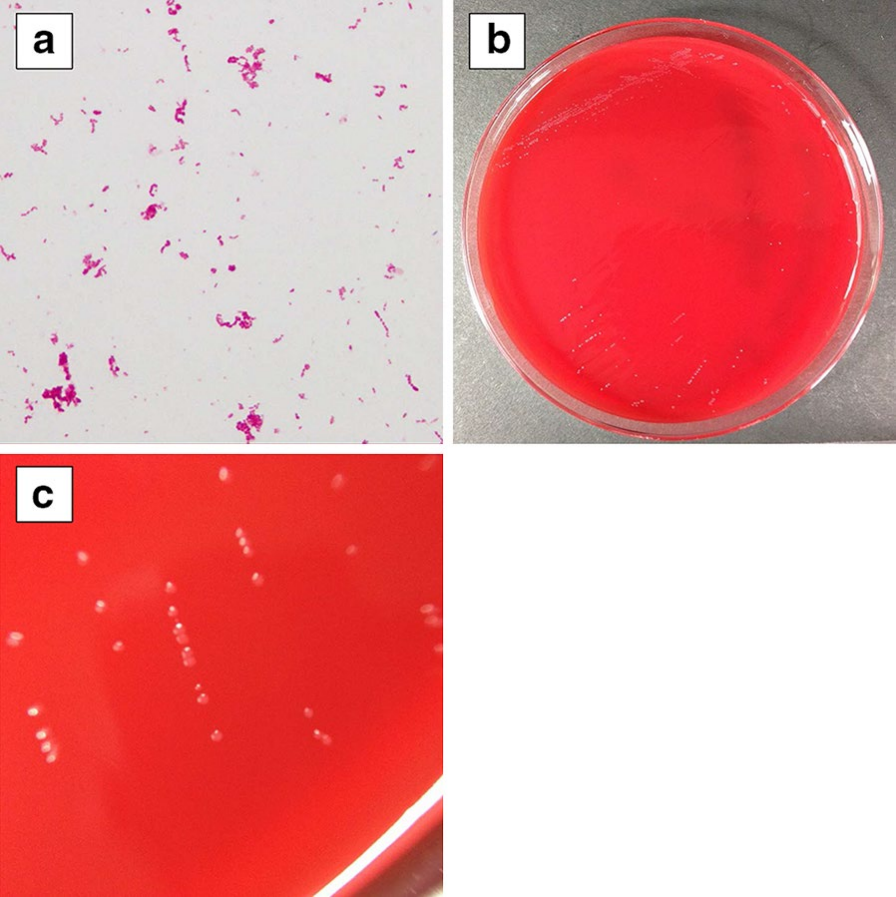
Microscopic images with Gram-staining (**a**). Highly pleomorphic, filamentous gram-negative bacilli can be seen in 2-day aerobic culture with 5 % CO_2_ on 5 % sheep blood agar. Colonies are very tiny, transparent and slightly white (**b**). High-power views of the colonies (**c**)

On the 2nd hospital day, highly pleomorphic, filamentous gram-negative bacilli were isolated from anaerobic blood culture of the clinical sample obtained on the day of admission (Fig. 1a). Isolates grew on both Brucella with Hemin and Vitamin K1 (HK) agar (Kyokuto Pharmaceutical Industrial Co., Ltd., Tokyo, Japan) and 5 % sheep blood agar (Eiken Chemical Co., Ltd., Tokyo, Japan) under both aerobic and anaerobic conditions. Those samples could be subcultured at 37 °C on 5 % sheep blood agar plates (Eiken Chemical Co., Ltd.) in a capnophilic atmosphere containing 5 % CO_2_ (Fig. 1b, c) according to previous reports [6, 7]. We obtained a colony approximately 1 mm in diameter in 3 days using these media. Though the isolate was not identified by biological techniques, we accurately identified the strain with 16S ribosomal ribonucleic acid (rRNA) genotyping, as previously described [8, 9], and a similarity search was conducted using the EzTaxon (http://www.ezbiocloud.net/eztaxon/). The isolate (GenBank accession no. LC062896) showed 100 % (1455/1455) similarity to the *Streptobacillus moniliformis* type strain DSM 12112^T^ (GenBank accession no. CP001779). A phylogenetic tree containing all available 16S rRNA sequences was constructed from a multiple sequence alignment with the neighbor-joining method using Molecular Evolutionary Genetics Analysis (MEGA) software, version 5.1 (Additional file 1: Figure). We performed antimicrobial susceptibility tests on the isolate employing both the microdilution method using MicroScan^®^ Negacombo 3.12 J (SIEMENS Inc. Berlin, Germany) and using lysed horse blood broth with microdilution, but we were unable to measure minimum inhibitory concentrations against several antibiotics, because there was no growth of the isolate even in the control well. Thus, though we switched the administered antibiotic to sulbactum/ampicillin (3.0 g/every 6 h) on the 2nd hospital day based on interim report of blood culture test, we had initially administered ampicillin (2.0 g/every 6 h) based on previous reports describing antimicrobial susceptibilities of similar isolates [2, 10].

**Fig. 2:**
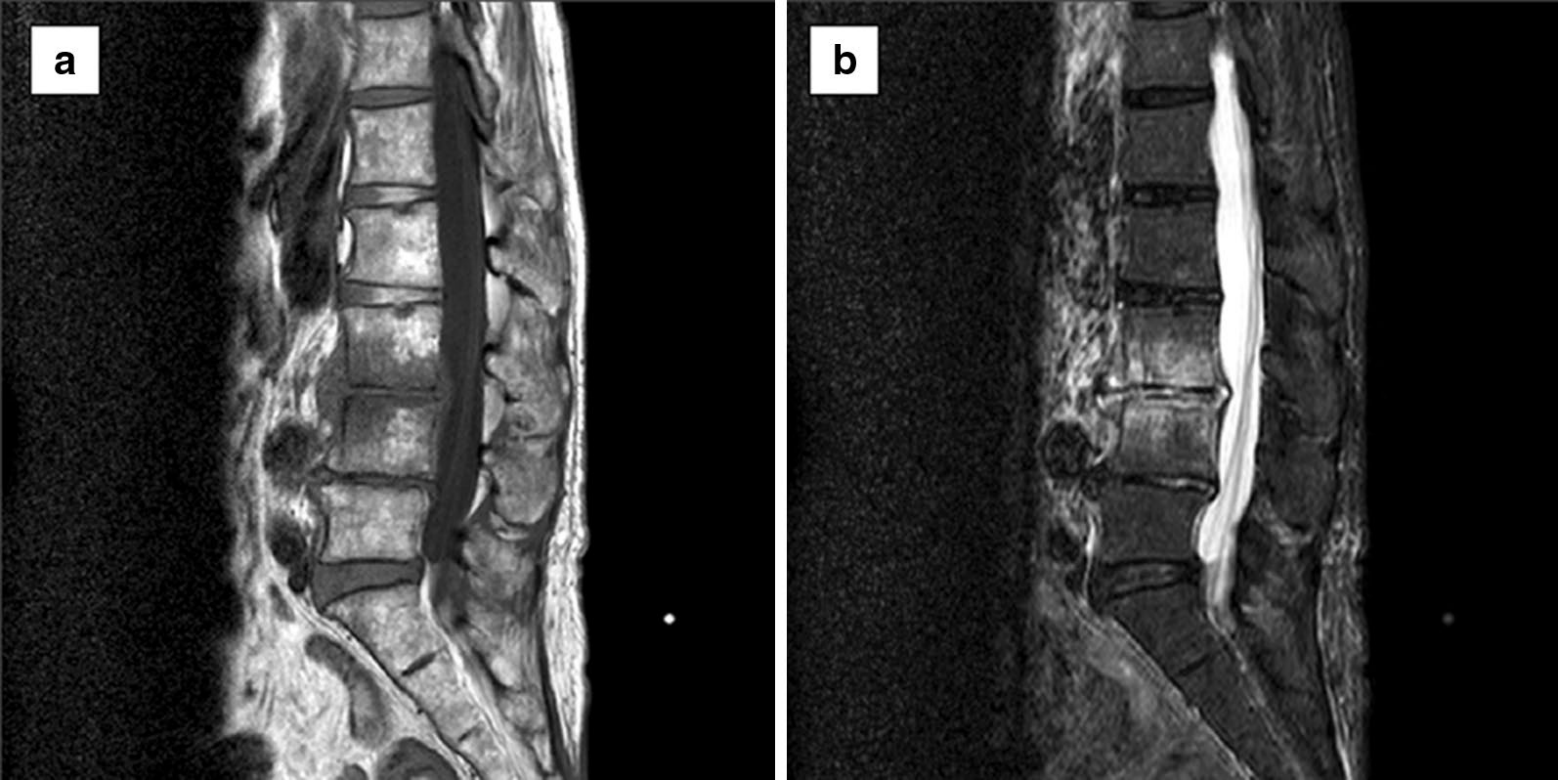
Magnetic resonance image of lumbar vertebrae obtained on the 10th hospital day. T1-weighted image (T1WI) and short T1 inversion recovery (STIR) T2 image (**a**, **b**), respectively. The lumbar sequence is linearized and the findings do not suggest a compression fracture, i.e. there is no dip-related transformation of the vertebral body. Vertebral bodies L3 and L4 have a low signal on T1WI and a high signal on STIR. The intervertebral disk is low and has a linear T2 high signal. We thus suspected this lesion to represent inflammation and/or degeneration

Though overall status including the fever showed improvement with antibiotic administration, the lower back pain persisted. She thus underwent magnetic resonance imaging (MRI) of the lumbar vertebrae. The MRI at the levels of the 3rd and 4th lumbar vertebrae showed a low signal on T1-weighted images (Fig. 2a), whereas signal intensity was high within the intranuclear cleft of the disk on short T1 inversion recovery (STIR) T2 imaging and disc extrusion with a high water content was observed adjacent to the extra-dural area (Fig. 2b). The vertebral endplates at this level were destroyed and high-signal-intensity bone marrow edema was visible. These findings are typical of pyogenic vertebral spondylodiscitis, but there were no signs of crush injury or transformation in the lesion. We continued the antibiotic administration and confirmed gradual improvement of her lower back pain. Furthermore, exercise capacity, including walking, gradually recovered. Follow-up MRI studies showed no deterioration of the lumbar lesions, and she was discharged on the 71st hospital day.

## Discussion

Rat bite fever has been a recognized zoonosis since ancient times in India, more than 2000 years ago. However, unexpectedly, this disease was reported again in the medical literature more recently. In 1840, Wilcox reported rat bite fever for the first time [11]. At the end of the 19th century, Miyake provided the first detailed description in the German literature [12]. In this report, he presented this disease as a definite clinical entity based on a series of 11 cases in his own experiences and prior references in the Japanese literature describing *Sodoku*, which means rat poisoning in Japanese.

*Streptobacillus moniliformis*, present in some rodent intraoral resident floras, is an aerobic or facultative anaerobic gram-negative, highly pleomorphic, filamentous, nonmotile, and non-acid-fast rod [2]. *S. moniliformis*, as well as *Spirillum minus*, transmitted by bite wounds and scratches from rodents such as mice, can cause systemic rat bite fever. This condition is due to infection by *S. moniliformis* or, less commonly, by *S. minus*. Disease caused by *S. minus* is generally known as *Sodoku* and occurs primarily in Asia [2]. After the incubation period of 2–10 days, symptoms progress to high fever, arthralgia of multiple joints, muscle ache, and whole body rash [1, 13–16]. In previous reports, arthritis occurred mainly in large joints including the elbows, knees and mid-back, while rashes of the palms or soles and were erythematous [3, 13, 14, 16–19]. Such skin lesions were occasionally accompanied by abscess formation [2]. On the other hand, *S. moniliformis* was reported to be a causative pathogen of infectious endocarditis [1, 20–24] or to be associated with immunocompromised status including human immunodeficiency virus (HIV) infection and hematological malignancies [24–26]. Furthermore, there are reports describing occurrence in patients with bacteremia associated with exposure [27, 28], such as working in laboratories [29, 30].

In the present case, fortunately, we were able to identify the causative organism from blood cultures. Thus, we provided appropriate treatment and were able to discharge the patient. An important point is whether she had suffered a rodent bite, but no such bite episode was revealed, despite a detailed interview. There are actually “unusual” case reports without a bite history [31] or febrile episode [5]. However, despite the lack of a clear bite history, we suspected *S. moniliformis* infection based on other aspects of the patient’s presentation.

She ran a dining room serving *Tonkatsu*, a Japanese dish which consists of a breaded, deep-fried pork cutlet, in Akihabara, an area of downtown Tokyo, and she had once seen a rat in the dining room kitchen. We considered the possibility of contracting the infection solely by contact with contaminated water and/or food. In a previous report, an outbreak of *S. moniliformis* blood stream infections occurred in a boarding school and the authors concluded the vehicle of infection to be rats contaminating the water supply [32]. Indeed, *S. moniliformis* infection is caused not only directly by rodent bites but also via contaminated water.

As for major changes in the global environment, such as deforestation and clearing for farmland as well as global climate change due to the greenhouse effect caused by the rise in carbon dioxide concentration, the risk of zoonoses will increase worldwide. However, epidemiological analysis will be difficult even if zoonosis increases, because accurate diagnosis of this disease is often difficult. The anticoagulant included in the commercial blood culture bottle reportedly inhibits the growth of *S. moniliformis*, and there are numerous cases lacking an accurate diagnosis [14, 33, 34]. In cases with a bite history, rat bite fever is really suspected, but in those without a bite history, the diagnosis must always be based on microbiological testing alone. An accurate diagnosis cannot be obtained without an isolate. A culture test and the improvement of the device for detecting *S. moniliformis* are highly anticipated.

## Conclusions

We conclude that *S. moniliformis* infection can become established with contaminated vehicle contact alone, not only as a direct result of a bite. Those working in places where rodents breed or are at risk of contact with rats or mice might be at risk for contracting this unusual disease. The cultural diversification of large urban centers is becoming increasingly complex. Thus, we must keep in mind the possibility of zoonosis.

## Consent

Written informed consent was obtained from the patient for publication of this Case Report and any accompanying images.

## Additional file

10.1186/s13104-015-1642-6 A phylogenetic tree containing all available 16S rRNA sequences.

## Abbreviations

RA: rheumatoid arthritis
rRNA: ribosomal ribonucleic acid
MRI: magnetic resonance imaging
STIR: short T1 inversion recovery

## References

[1] Madhubashini M, George S, Chandrasekaran S (2013). Streptobacillus moniliformis endocarditis: case report and review of literature. Indian Heart J.

[2] Elliott SP (2007). Rat bite fever and *Streptobacillus moniliformis*. Clin Microbiol Rev.

[3] Dendle C, Woolley IJ, Korman TM (2006). Rat-bite fever septic arthritis: illustrative case and literature review. Eur J Clin Microbiol Infect Dis.

[4] Wullenweber M (1995). *Streptobacillus moniliformis*–a zoonotic pathogen. Taxonomic considerations, host species, diagnosis, therapy, geographical distribution. Lab Anim.

[5] Stehle P, Dubuis O, So A, Dudler J (2003). Rat bite fever without fever. Ann Rheum Dis.

[6] Woo PC, Wu AK, Tsang CC, Leung KW, Ngan AH, Curreem SO, Lam KW, Chen JH, Chan JF, Lau SK (2014). Streptobacillus hongkongensis sp. nov., isolated from patients with quinsy and septic arthritis, and emended descriptions of the genus *Streptobacillus* and *Streptobacillus moniliformis*. Int J Syst Evol Microbiol.

[7] Eisenberg T, Glaeser SP, Nicklas W, Mauder N, Contzen M, Aledelbi K, Kämpfer P (2015). Streptobacillus felis sp. nov. isolated from a cat with pneumonia. Int J Syst Evol Microbiol.

[8] Nei T, Akutsu K, Shima A, Tsuboi I, Suzuki H, Yamamoto T, Tanaka K, Shinoyama A, Kojima Y, Washio Y, Okawa S, Sonobe K, Norose Y, Saito R (2012). A case of streptococcal toxic shock syndrome due to Group G streptococci identified as Streptococcus dysgalactiae subsp. equisimilis. J Infect Chemother.

[9] Nei T, Hyodo H, Sonobe K, Dan K, Saito R (2012). First report of infectious pericarditis due to Bordetella holmesii in an adult patient with malignant lymphoma. J Clin Microbiol.

[10] Edwards R, Finch RG (1986). Characterisation and antibiotic susceptibilities of *Streptobacillus moniliformis*. J Med Microbiol.

[11] Wilcox M (1840). Violent Symptoms from the Rat Bite. Am J Med Sci.

[12] Miyake H (1899). Ueber Die Rattenbisskrankheit. Mittelungen aus den Grenzgebieten der Medizin und Chirurgie..

[13] Flannery DD, Akinboyo I, Ty JM, Averill LW, Freedman A (2013). Septic arthritis and concern for osteomyelitis in a child with rat bite fever. J Clin Microbiol.

[14] Orlev A, Miskin I, Temper V, Korem M (1007). A 70-year-old man with fever and polyarthralgia. *Streptobacillus moniliformis*. Clin Infect Dis.

[15] Ojukwu IC, Christy C (2002). Rat-bite fever in children: case report and review. Scand J Infect Dis.

[16] Hockman DE, Pence CD, Whittler RR, Smith LE (2000). Septic arthritis of the hip secondary to rat bite fever: a case report. Clin Orthop Relat Res.

[17] Budair B, Goswami K, Dhukaram V. Septic arthritis secondary to rat bite fever: a challenging diagnostic course. BMJ Case Rep. 2014. doi:10.1136/bcr-2014-204086. 10.1136/bcr-2014-204086 PMC3987209 24695665

[18] Dworkin J, Bankowski MJ, Wenceslao SM, Young R (2010). A case of septic arthritis from rat-bite fever in Hawai’i. Hawaii Med J.

[19] Vasseur E, Joly P, Nouvellon M, Laplagne A, Lauret P (1993). Cutaneous abscess: a rare complication of *Streptobacillus moniliformis* infection. Br J Dermatol.

[20] Fenn DW, Ramoutar A, Jacob G, Bin Xiao H. An unusual tale of rat-bite fever endocarditis. BMJ Case Rep. 2014. doi:10.1136/bcr-2014-204989. 10.1136/bcr-2014-204989 PMC4244522 25414213

[21] Chen PL, Lee NY, Yan JJ, Yang YJ, Chen HM, Chang CM (2007). Prosthetic valve endocarditis caused by *Streptobacillus moniliformis*: a case of rat bite fever. J Clin Microbiol.

[22] Kondruweit M, Weyand M, Mahmoud FO, Geissdörfer W, Schoerner C, Ropers D, Achenbach S, Strecker T (2007). Fulminant endocarditis caused by *Streptobacillus moniliformis* in a young man. J Thorac Cardiovasc Surg.

[23] McCormack RC, Kaye D, Hook EW (1967). Endocarditis due to *Streptobacillus moniliformis*. JAMA.

[24] Rordorf T, Züger C, Zbinden R, von Graevenitz A, Pirovino M (2000). *Streptobacillus moniliformis* endocarditis in an HIV-positive patient. Infection.

[25] Chean R, Stefanski DA, Woolley IJ, Francis MJ, Korman TM (2012). Rat bite fever as a presenting illness in a patient with AIDS. Infection.

[26] De AS, Baveja SM, Salunke PM, Manglani MV (2010). Isolation of Streptobacillus moniliformis from the blood of a child with acute lymphoblastic leukaemia. Indian J Med Microbiol.

[27] Wilkins EG, Millar JG, Cockcroft PM, Okubadejo OA (1988). Rat-bite fever in a gerbil breeder. J Infect.

[28] Shvartsblat S, Kochie M, Harber P, Howard J (2004). Fatal rat bite fever in a pet shop employee. Am J Ind Med.

[29] Anderson LC, Leary SL, Manning PJ (1983). Rat-bite fever in animal research laboratory personnel. Lab Anim Sci.

[30] Hamburgar M, Knowles HC (1953). *Streptobacillus moniliformis* infection complicated by acute bacterial endocarditis; report of a case in a physician following bite of laboratory rat. AMA Arch Intern Med.

[31] Fordham JN, McKay-Ferguson E, Davies A, Blyth T (1992). Rat bite fever without the bite. Ann Rheum Dis.

[32] McEvoy MB, Noah ND, Pilsworth R (1987). Outbreak of fever caused by Streptobacillus moniliformis. Lancet.

[33] Shanson DC, Pratt J, Greene P (1985). Comparison of media with and without ‘Panmede’ for the isolation of *Streptobacillus moniliformis* from blood cultures and observations on the inhibitory effect of sodium polyanethol sulphonate. J Med Microbiol.

[34] Palarasah Y, Skjoedt MO, Vitved L, Andersen TE, Skjoedt K, Koch C (2010). Sodium polyanethole sulfonate as an inhibitor of activation of complement function in blood culture systems. J Clin Microbiol.

