# Supplementary material for: Streptobacillus moniliformis bacteremia in a rheumatoid arthritis patient without a rat bite: a case report
Source: BMC Res Notes. 2015 Nov 19;8:694. doi: 10.1186/s13104-015-1642-6 (PMC4653872; doi:10.1186/s13104-015-1642-6)
Supplement: Supplementary file 1 — 10.1186/s13104-015-1642-6 A phylogenetic tree containing all available 16S rRNA sequences. [file 13104_2015_1642_MOESM1_ESM.pptx]

## Slide 1
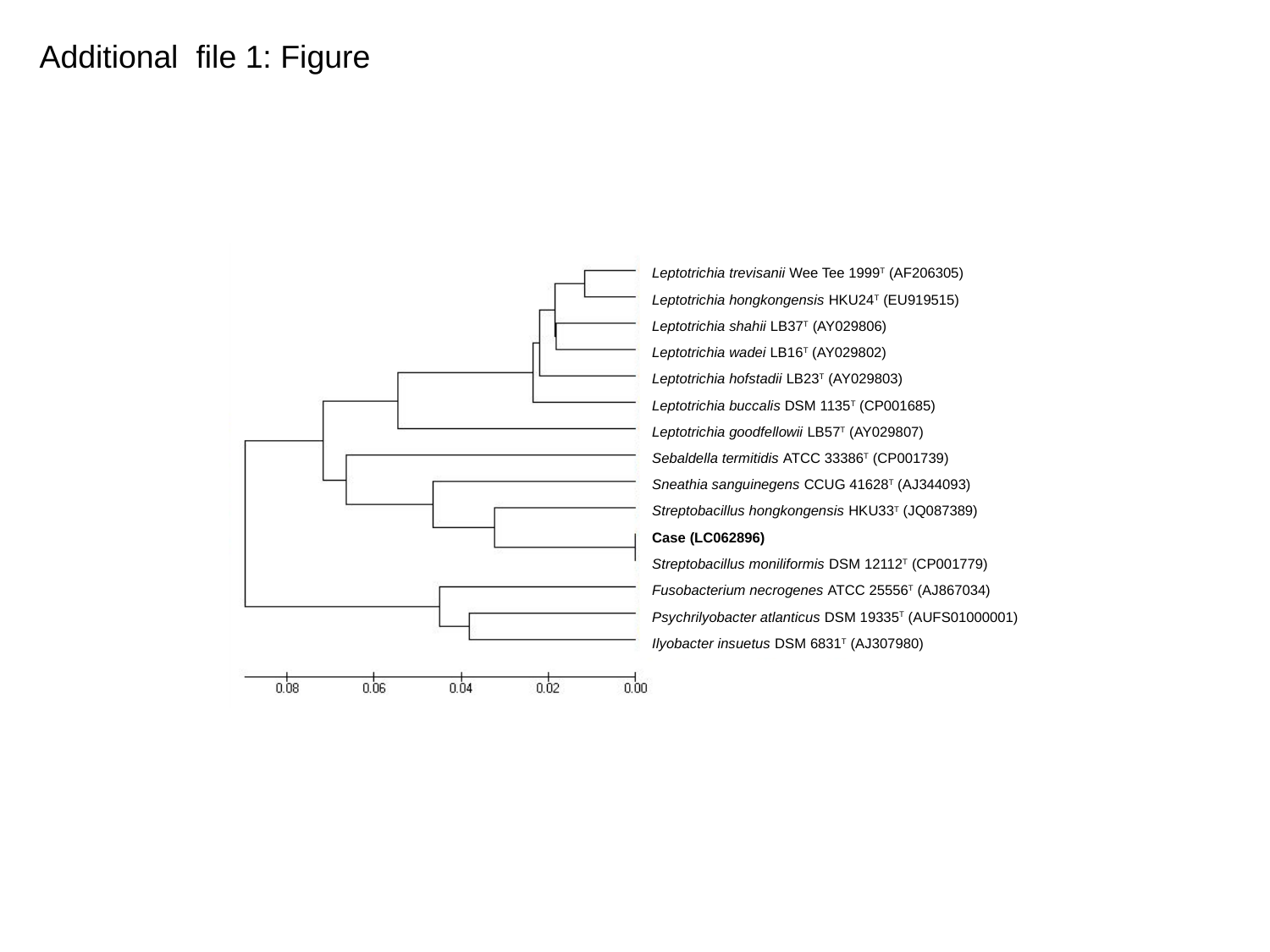

Additional file 1: Figure
Leptotrichia trevisanii Wee Tee 1999T (AF206305)
Leptotrichia hongkongensis HKU24T (EU919515)
Leptotrichia shahii LB37T (AY029806)
Leptotrichia wadei LB16T (AY029802)
Leptotrichia hofstadii LB23T (AY029803)
Leptotrichia buccalis DSM 1135T (CP001685)
Leptotrichia goodfellowii LB57T (AY029807)
Sebaldella termitidis ATCC 33386T (CP001739)
Sneathia sanguinegens CCUG 41628T (AJ344093)
Streptobacillus hongkongensis HKU33T (JQ087389)
Case (LC062896)
Streptobacillus moniliformis DSM 12112T (CP001779)
Fusobacterium necrogenes ATCC 25556T (AJ867034)
Psychrilyobacter atlanticus DSM 19335T (AUFS01000001)
Ilyobacter insuetus DSM 6831T (AJ307980)
